# Effects of extrinsic foot musculature on hindfoot kinematics during stance phase: Implications for flatfoot pathology

**DOI:** 10.1186/1757-1146-5-S1-O49

**Published:** 2012-04-10

**Authors:** Josefien Burg, Koen Peeters, Tassos Natsakis, Jos Vander Sloten, Greta Dereymaeker, Ilse Jonkers

**Affiliations:** 1 Mechanical Engineering Department, K.U.Leuven, Belgium; 2 Department of Kinesiology, K.U.Leuven, Belgium

## Background

Flatfoot deformity is a common condition, characterized by a collapse of the medial foot arch. Specific muscle dysfunctions relate to kinematic changes of the hind foot (plantarflexion, abduction and valgus) inducing the onset of flatfoot deformity. However, to determine a causal relation between individual muscle action, foot bone motion and flatfoot, in vitro experiments are needed. Our hypothesis states that inducing altered muscle forces in cadaveric feet causes alterations in kinematics, representative for flatfoot deformity [1,2].

## Materials and methods

A gait simulator was used to test seven cadaveric feet. Pneumatic actuators applied forces to the foot tendons, simulating flatfoot related pathologies: contracture of M.Triceps Surae (C-TS) and Mm.Peronei (C-PE); weakness of M.Tibialis Posterior (W-TP) and the pretibial muscles (W-PT); combined contracture of TS and PE (P1) and combined TS contracture with TP weakness (P2). Trajectories of bone-embedded LED clusters were measured during a one second roll-off and resulting ankle, subtalar and talonavicular joint motion was calculated.

## Results

At all joint levels, increased motion towards valgus and adduction is seen. Exceptions are subtalar abduction with C-PE and C-TS as well as ankle and subtalar varus for C-PE and P1. More variability is observed for plantar-dorsiflexion: all joints show plantarflexion with C-TS and W-PT, except for subtalar dorsiflexion with W-PT. Dorsiflexion appears with W-TP and C-PE.

## Conclusion

We can confirm the contribution of altered muscle action (either contracture or weakness) to flatfoot development. Our results suggest that largest contribution is present in frontal and sagittal plane, with hindfoot motion towards valgus and plantarflexion. No abduction was seen, suggesting lesser contribution of these pathologies in the transverse plane. This study contributes to fundamental knowledge on foot biomechanics in normal as well as pathological foot function and establishes the causal relation between modified muscle action and flatfoot deformity.

**Figure 1:**
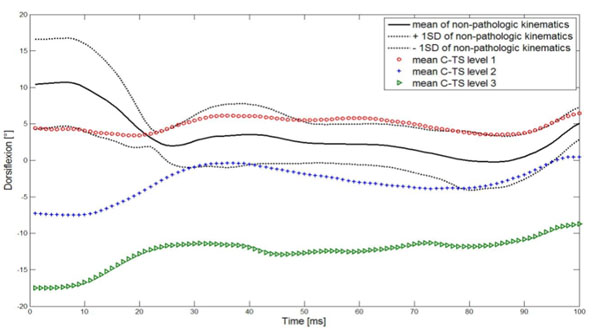
Kinematic changes at the ankle joint with three levels of TSC
